# Electrochemical Biosensors Based on Membrane-Bound Enzymes in Biomimetic Configurations

**DOI:** 10.3390/s20123393

**Published:** 2020-06-16

**Authors:** Julia Alvarez-Malmagro, Gabriel García-Molina, Antonio López De Lacey

**Affiliations:** Instituto de Catálisis y Petroleoquímica, CSIC, c/Marie Curie 2, 28049 Madrid, Spain; j.malmagro@csic.es (J.A.-M.); gabriel.garcia.m@csic.es (G.G.-M.)

**Keywords:** biosensor, biomimetic membranes, membrane-bound enzymes, electrodes

## Abstract

In nature, many enzymes are attached or inserted into the cell membrane, having hydrophobic subunits or lipid chains for this purpose. Their reconstitution on electrodes maintaining their natural structural characteristics allows for optimizing their electrocatalytic properties and stability. Different biomimetic strategies have been developed for modifying electrodes surfaces to accommodate membrane-bound enzymes, including the formation of self-assembled monolayers of hydrophobic compounds, lipid bilayers, or liposomes deposition. An overview of the different strategies used for the formation of biomimetic membranes, the reconstitution of membrane enzymes on electrodes, and their applications as biosensors is presented.

## 1. Introduction

The attachment of enzymes to electrodes has been in the last decades a powerful strategy for the development of efficient biosensors. It couples the high specificity and turnover of enzymatic catalysis, thus assuring selective target detection and signal amplification, with the versatility, fast-response, sensitivity, and simplicity of electrochemical transduction [1,2].

Although a high proportion of redox enzymes in nature are membrane-bound ones, either associated to the external part or trans-membrane, most of the developed enzymatic biosensors are based on soluble ones. The main reasons are the higher structural complexity and lower stability of the purified membrane-bound enzymes. In fact, in most electrochemical studies that involve membrane-bound enzymes, their hydrophobic subunits are dissociated, which generally results in diminished activity and stability [3]. In order to take full advantage of the use of membrane-bound enzymes in electrochemical devices, it is necessary to design immobilization procedures that favor maintaining their natural configuration by stabilizing their hydrophobic regions. The formation of biomimetic membranes over electrode surfaces that are based on phospholipid bilayers is a versatile and powerful option for the reconstitution of membrane-bound enzymes [4,5]. Other methods that have been successfully used for developing electrochemical biosensors based on this type of enzymes have been the deposition of liposomes or formation of monolayers of hydrophobic compounds on the electrode surface [6,7].

In this review, we provide an overview of the strategies developed for the reconstitution of membrane-enzymes on biomimetic membranes over electrodes and, in particular, focusing on works that aimed at their application as electroenzymatic biosensors.

## 2. Biomimetic Membranes on Electrodes

Lipid biomimetic membranes that formed on solid materials are the key to model the properties of cell membrane processes, opening new research opportunities for surface electrochemistry [8]. These architectures provide simple systems, where it is possible to study in a systematically way fundamental membrane-related processes while preserving the essential characteristics of the membrane, such as fluidity or electrical sealing properties [9]. Indeed, gold substrates show a particular interest, because it is a common material that is employed for biomedical devices, such as electrochemical biosensors.

**Figure 1:**
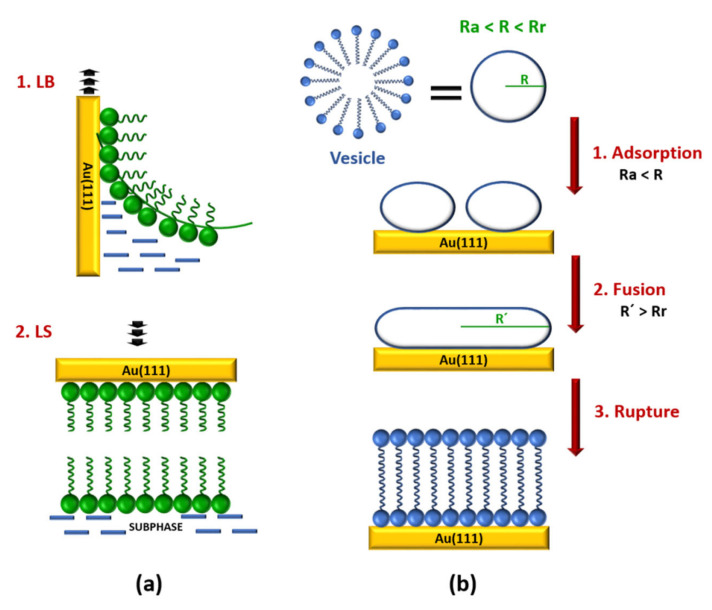
Schematic representation of the preparation of lipid bilayers on gold substrates by (**a**) a combination of Langmuir–Blodgett and Langmuir–Schaefer (LB-LS) methods and (**b**) vesicle fusion (VF). Adapted with permission from [11,13]. Copyright (2000 and 2007) American Chemical Society.

Lipid bilayers over gold surfaces are normally built by combination of Langmuir–Blodgett (LB) and Langmuir–Schaefer (LS) transfer methods or by vesicle fusion (VF) [10]. The LB technique (Figure 1a) allows for forming a lipid monolayer on gold substrates [11]. In a typical LB deposition, the gold electrode is immersed in a water subphase under an air/water interphase, in which a lipid solution in chloroform is spread. After the evaporation of the chloroform a lipid monolayer is formed in the air/water interphase. Subsequently, the monolayer is slowly compressed to a controlled surface pressure. Finally, the deposition is accomplished by raising the gold substrate from the subphase through the compressed monolayer. The subphase pressure of the monolayer should be in the range from 10 to 40 mN m^−1^, and the monolayer temperature needs to be controlled, ensuring that the organic film is in a condensed and stable state, to obtain a good transfer ratio (τ) between 0.9 and 1.1. The microstructure and the packing density of this monolayer (the inner layer) affects the structure of the successive monolayer (the outer layer) deposited on the already modified gold electrode [12]. Drying completely the first transferred monolayer has been shown to improve the deposition process of the second layer [10]. The outlet layer of the bilayer is deposited by the LS technique (Figure 1a) [11]. The substrate horizontally touches a compressed lipid monolayer in a subphase and it is immediately withdrawn slowly. As a result, a Y-type bilayer, in which the membrane is in tail-to-tail arrangement, on the gold substrate is obtained. Because the deposition of each layer is an independent process, the LB–LS deposition method allows for constructing asymmetric bilayers. Moreover, this strategy produces more stable and better ordered bilayers.

The VF method is an easier strategy for preparing bilayers that include integral membrane proteins from proteoliposomes [13,14]. A vesicle is a biological structure that consists of liquid enclosed by a lipid bilayer. Briefly, the VF process on gold substrates involves several steps (Figure 1b). Firstly, small unilamellar vesicles (20–50 nm in diameter), which are in an aqueous vesicle dispersion, are adhered to the substrate in highly ordered stripe-like domains. Subsequently, the fusion takes place giving a hemimicelle film. As a result, a lipid bilayer is formed by rupturing of the vesicles, unrolling, and spreading onto gold substrate. It is assumed that all of these steps depend on the vesicle size [12,13,15,16]. The VF process requires that the initial vesicle radius (R) is higher than the critical adsorption radius (Ra) and lower that the minimum rupture radius (Rr). If the radius of the vesicle is lower than Ra, the adsorption process does not take place, whereas, if the radius is higher than the Rr, then the vesicle can rupture and directly form a single bilayer disk [13]. However, for a successful rupturing of the adhered vesicles in the second step the new vesicles radius (R´) need to be higher than Rr. Moreover, temperature, presence of cations, surface charge, surface roughness, ionic strength, and solution pH should be also taken into account to obtain a good bilayer [17]. Alternatively, if gold electrodes are previously modified with relatively low hydrophobic self-assembled monolayers (SAMs) a lipid bilayer can be built by the rapid solvent exchange technique reported by Cornell et al. [18]. In this method, the SAM-coated gold electrode is incubated in a lipid solution in ethanol, followed by a fast transfer to an aqueous buffer solution. As a result, more reproducible bilayers with less defects are obtained.

**Figure 2:**
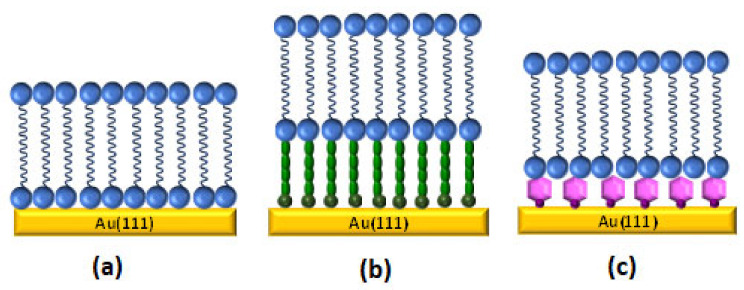
Cartoon of some type of biomimetic membranes at gold surface: (**a**) supported bilayer lipid membranes (sBLM), (**b**) tethered bilayer lipid membranes (tBLM), and (**c**) floating bilayer lipid membranes (fBLM). Adapted from [19] with permission from Elsevier.

The use of gold electrodes as substrate allows for obtaining molecular level of information from the bilayer formed on top. In the recent years, a combination of traditional electrochemical techniques and surface sensitive methods, such as spectroscopy, neutron scattering, and microscopic methods, have been employed to understand the behavior and structure of these biomimetic systems. On gold surfaces, it is possible to build different types of biomimetic bilayers (Figure 2), such as metal supported bilayer lipid membranes (sBLMs), tethered bilayer lipid membranes (tBLMs), or floating bilayer lipid membranes (fBLMs) [19,20].

Metal supported lipid membranes (sBLMs) (Figure 2a) were the first generation of lipid bilayer system used to mimic and understand the cell membrane processes. In the sBLMs, the inner layer is directly adsorbed onto the gold substrate [12]. Lipkowski’s group was pioneer in successfully building sBLMs on gold electrodes either by VF [21,22,23] or a combination of LB-LS techniques [11,24]. The quality of the bilayer can be improved by the addition of 30% of cholesterol. The lipid membrane becomes more fluid and stress within the membrane is released, which is closer to a real biological cell membrane [25]. Atomic force microscopy (AFM) [16], scanning tunneling microscope (STM) [16,26,27], and electrochemical scanning tunneling microscope EC-STM [28] techniques have been employed to obtain molecular imaging information and molecular level resolution about the electrode surface. sBLMs on gold electrodes behave as an ideal capacitor over a large potential range. The effect of the electric field has effectively been studied with fundamental electrochemical techniques, such as differential capacitance and charge density. These results allow for the characterization of sBLMs providing information regarding the quality, compactness, and defect level of supported bilayer lipid membranes [19]. Electrochemical impedance spectroscopy (EIS) analysis provides interesting quantitative information about the interphase. A model that considers the sBLMs systems, like a series of constant phase elements (CPE) and an ohmic resistance, was employed to simulate the electrical properties of the interphase for a better understanding of interfacial processes, such as the incorporation of a carrier into real cell membranes and drug release [29,30]. In sBLMs, each side of the bilayer is exposed to different environments. The outer layer is exposed to the electrolyte while the inner leaflet is physically adsorbed on the gold subphase. From this point of view, electrochemical measurements provide valuable information regarding average properties, such as the effect of the electric field in the orientation and conformation in each layer of the membrane. However, it is not possible to obtain insight into the structure of the membrane at molecular level. The polarization modulation infrared reflection absorption spectroscopy (PM-IRRAS) technique, developed in Lipkowski´s group [31], has been successfully employed to evaluate the structure and organization of sBLMs. It demonstrated that the inner leaflet is more ordered than the outer leaflet [24], which agree with the physical and kinetic properties of the membrane constituents [32,33]. sBLMs have been used as a model system to study biological processes, like peptide incorporation [34], ligand-receptor interactions [35], or drug delivery process [36]. However, studies involving the inclusion of large transmembrane proteins are not possible with sBLM, because a water reservoir (1–2 nm thick water-rich layer) is needed between the bilayer and the gold electrode to avoid denaturation or the alteration of the functionality of the proteins [32,37].

To address this issue, tethered bilayer lipid membranes (tBLMs) (Figure 2b), where the bilayer is covalently bound to the gold electrode via hydrophilic tethering molecules [38], have been employed. These platforms show high mechanical and chemical stability. Traditionally, a tBLM is built in two separated steps [39]. First, the inner layer is self-assembled through a covalent bond with the gold electrode. This layer usually consists on flexible disulphide or thiolipid derivatives with a hydrophilic part [9] attached to the electrode by a covalent Au-S bond. The hydrophilic part provides an ion and water reservoir underneath the bilayer. In the second step, the outer layer is anchored by VF [9,40] or rapid solvent exchange [41]. Alternatively, the composition of the lipid tethered bilayer can be modified by vesicle exchange [42]. PM-IRRAS [43], neutron reflectivity experiments [44], and surface enhanced infrared absorption spectroscopy (SEIRAS) [45] have been used to evaluate the densely packed state of the SAM component, as well as the relation with the amount of water present between the bilayer and the electrode. These studies predicted that the insertion of protein and peptides is more favorable into sparsely than densely packed tBLMs [45]. In terms of electrical properties, tBLMs are stable model membrane systems [18,31,46]. EIS provides unique information regarding the electrical parameters of these biomimetic membranes. A model that was developed by Valincius et al. considering membrane defects has been employed to evaluate changes in capacitance and membrane resistance induced by the incorporation of protein or peptide molecules in the bilayer [47,48]. AFM imaging has allowed for determining the surface morphology of the tBLMs [9] as well as to provide visual images concerning the insertion of the protein or peptide [48]. Despite all of the advantages when compared to sBLMs, tBLMs still do not display long term stability and present a restrictive mobility. These are two important disadvantages to employ these platforms for biosensors development.

Floating bilayer lipid membranes (fBLMs) (Figure 2c), which interact with the gold substrate by physical interactions, are able to mimic the quasi natural environment of real membranes. They show better lateral mobility of the bilayer [48] and reduce the risk of protein denaturalization, thus preserving it activity. fBLMs are composed by a bilayer that floats ∼2.4 nm over a supporting layer on the surface [21,49] or a monolayer of S-layer protein [50]. The inner leaflet should be a water rich lubricant layer that usually is a water rich polymer [16,51,52,53] or a hydrogel film [54]. The outer bilayer is deposited by VF [45] or a combination of LB-LS [37,55]. AFM images [37] confirmed that the lipid molecules in fBLM are tightly packed. Moreover, PM-IRRAS studies demonstrated that the lipid bilayer in fBLMS is separated from the gold surface by a water region, which allows for packing the lipid in a zigzag configuration [55]. Recently, SEIRAS [56] has been employed to probe that water molecules are a more ordered structure in the sub-membrane region of a fBLM than in a bulk solution. This strategy is a potential tool for obtaining the molecular level of information related with the hydration of fBLM and with the changes induced when a protein is incorporated.

## 3. Reconstitution of Membrane-Bound Enzyme on Electrodes

Reconstituted membrane enzymes play an important role in several fields, such as medicine, analytical chemistry, alternatives energies, and materials development [57]. One of the main problems for the application of these enzymes is their denaturation and loss of catalytic activity when they are not in the native-like environments [3]. Coupling the catalytic function of membrane enzymes to an electrode requires the optimization of their immobilization process, so that their in vivo structure is preserved. The best strategies for this purpose involve creating a biomimetic environment of the membrane enzyme that is attached to the electrode surface. Electronic communication between the enzymatic active site and the electrode can then be established by direct electron transfer (DET) or through the incorporation of a redox compound for achieving mediated electron transfer (MET).

**Figure 3:**
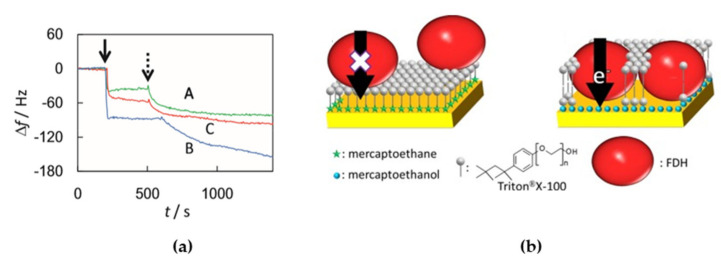
(**a**) Frequency changes measured by Quartz Crystal Microbalance (QCM) on the addition of 1% Triton^®^ X-100 (at the solid arrow) and fructose dehydrogenase (FDh) (at the dashed arrow) at mercaptoethane (MEtn)-modified (green line), 2-mercaptoethanol (MEtOH)-modified (blue line), and bare Au electrodes (red line). (**b**) Proposed scheme of the adsorption of fructose dehydrogenase (FDh) and Triton^®^ X-100 to hydrophobic (left) and hydrophilic electrodes (right). Reprinted from [61] with permission from Elsevier.

Membrane enzymes have high structural complexity, thus both electrostatic and hydrophobic forces contribute greatly during the processes of adsorption onto electrodes and the subsequent electronic transfer. When the enzyme has certain surface regions in which charged amino acid zones predominate, then the adequate modification of the electrode surface can modulate the orientation of the immobilized enzyme molecules [58,59,60]. As this kind of enzymes have hydrophilic and hydrophobic domains, surfactants are necessary for the solubility of the membrane enzymes after their purification. It has to be taken into account that the surfactant might adsorb to the electrode, therefore affecting DET or even suppressing it completely [61]. Kawai et al. studied the effects of Triton^®^ X-100 (a non-ionic surfactant) on the DET process of membrane-bound formate dehydrogenase (FDh) from *Gluconobacter japonicus* adsorbed onto gold electrodes modified with different thiol SAMs [61]. Changes in the frequency observed in Quartz Crystal Microbalance (QCM) measurements, performed in order to monitor the adsorption of Triton^®^ X-100 over the SAMs, showed the formation of a surfactant monolayer (−40 Hz) in the electrodes modified with mercaptoethane (MEtn) and a bilayer (−100 Hz) in the case of 2-mercaptoethanol (MetOH) (Figure 3a). The surfactant monolayer interacted strongly over the hydrophobic MEtn SAM, thus preventing DET of the adsorbed FDh, which would have its redox centers to far away from the electrode surface. Under the same conditions, but with a hydrophilic MetOH SAM on the electrode surface, the surfactant formed a bilayer over the SAM interacting very weakly with it and allowed the insertion of FDH into the surfactant bilayer for DET with the electrode (Figure 3b). 

On the other hand, Lojou and co-workers found that remains of the detergent n-Dodecyl β-D-maltoside (DDM) strongly attached around the hydrophobic zones surrounding the distal 4Fe4S cluster (the redox site for electron exchange) of the membrane-bound NiFe hydrogenase (Hase) from *Aquifex aeolicus* when adsorbed on modified gold electrodes. This effect modifies the hydrophobicity of this areas, which makes them more hydrophilic. Therefore, in the case of the enzyme adsorption on an electrode modified with hydrophobic SAMs, the enzyme molecules always oriented with the distal cluster region on the opposite side to the SAM, too far for establishing DET with the electrode. In the case of hydrophilic SAMs on the electrode, there was no preferential enzyme orientation during adsorption, so the MET and DET processes had the same incidence with a catalytic current ratio of I_DET_/I_DET+MET_ around 0.5 for H_2_ oxidation [59].

Cytochrome p450 (CyP) and human flaving containing monooxygenase 3 (hFMO3) are membrane-bound redox enzymes that have also been studied for optimizing their DET with electrodes modified with hydrophobic SAMs. In the case of the CyPs, its active center is an iron protoheme. The natural compounds that supply electrons to CyPs for their catalytic activity, NADPH, is very expensive and time consuming. Immobilizing CyPs on electrodes can replace these products [62]. Microsomes (lipid membranes) containing CyP and CyP reductase (CPR) and deposited on electrodes modified with hydrophobic SAMs of aromatic compounds benzenethiolate (BT) and naphtalene thiolate (NT) gave good current intensities by DET with reduction peaks around −0.4 V vs Ag/AgCl. The electroenzymatic system was tested for testosterone metabolization, measuring by high performance liquid chromatography (HPLC) a production of 270 pmol of 6 β-hydroxytestosterones [63]. hFMO3 is a liver protein that belongs to the second most important class of phase-1 drug-metabolizing enzymes [64,65,66]. Castrignano et al. reported the immobilization of hFMO3 on glassy carbon/graphite oxide (GO) modified with di-dodecyl di-methylammonium bromide (DDAB), which mimicked the enzyme´s native environment. By HPLC, they measured the products obtained from the electroenzymatic N-oxidation of benzydamine (a nonsteroidal anti-inflamatory) and tamoxifen (an antiestrogenic used in therapies against breast cancer and chemoprotection) [66].

**Figure 4:**
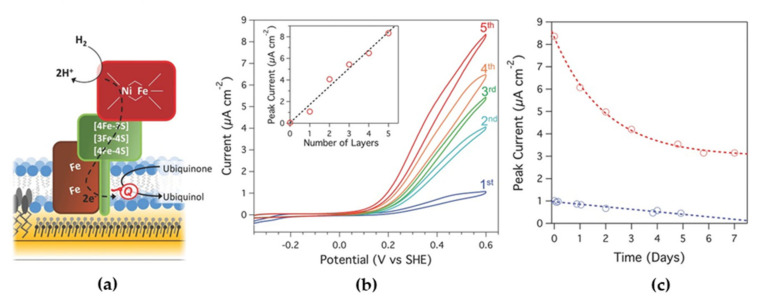
(**a**) Scheme of immobilized MBH activity on a biomimetic membrane-modified electrode. Ubiquinone is reduced by the enzyme to ubiquinol using electrons generated from hydrogen oxidation. The ubiquinone is reoxidized at the electrode. (**b**) Cyclic voltammograms (CVs) for 1–5 lipid bilayers containing MBH. Inset: Catalytic current (measured at 600 mV) as a function of the number of lipid bilayers. (**c**) Peak current (obtained at 600 mV) as a function of time (up to a week) for a five-layered MBH multilayer (red) and a single-MBH bilayer (blue) as measured by CV. Reproduced from [68] under the terms and conditions of the Creative Commons Attribution (CC BY) license.

Quinone oxidoreductases are a type of membrane-bound enzymes that catalyze redox processes of the quinone pool in cell membranes. They can be reconstituted on sBLMs formed over electrodes, in which lipophilic quinones that are embedded in the sBLM act as redox mediators with the electrode [67]. Jeuken and co-workers studied this strategy for an ubiquinol oxidase (cytochrome bo3 from *Escherichia coli*), which couples the oxidation of ubiquinol to ubiquinone with the reduction of O_2_ to H_2_O [68]. Furthermore, they used the layer by layer (LBL) technique with bacterial membrane extracts on gold electrodes to create multilayers of ubiquinol oxidase. In this strategy, poly-l-lysine was used as an electrostatic polymer for connecting the different lipid bilayers. The same strategy was studied with an oxygen tolerant membrane bound Hase (MBH, Figure 4a). This type of NiFe-Hases have has three subunits, one of them is a hydrophobic one that is inserted in the cell membrane and ensures the electron transfer between the other Hase subunits and the pool of quinones of the respiratory chain [69]. Measurements by fluorescence recovery after bleaching were performed to determine the lateral diffusion for the base bilayer and for the interconnections between bilayers, being 0.6 ± 0.1 µm^2^ s^−1^ and 0.7 ± 0.2 µm^2^ s^−1^, respectively [68]. Thus, these values are indistinguishable from each other, indicating that the membrane stacks are interconnected via lipid phases. These interconnections can be maintained throughout the layers, creating diffusion routes throughout the multilayer, not only of lipids, but also of lipophilic quinones within the bilayer, such as ubiquinone-10 (UQ10) and menoquinone-7 (MQ7). An important property of quinones is their hydrophobicity, which restricts them within the bilayer, but also allows them to diffuse freely within it, allowing for MET-based electoenzymatic catalysis. The cyclic voltammograms (CVs) performed showed a linear increase in the MQ7 current and in its peak area with the number of lipid bilayers deposited on the electrode, indicating an increase in the number of quinones that interact with the electrode. The same behavior was observed for UQ10 [68]. In the CVs, electrocatalytic H_2_ oxidation coincided with the ubiquinol oxidation peak, confirming that the electronic transfer between MBH and the electrode was mediated by the quinone pool (Figure 4b), with a considerable stability of the immobilized membrane enzyme (Figure 4c). 

Another Hase, the NiFeSe one from *Desulfovibrio vulgaris*, is attached to the periplasmic cell membrane in a different way to that of the O_2_-tolerant Hases. Instead of having a hydrophobic third subunit inserted into the membrane, it is peripherally attached via a lipid tail at its N-terminus, which is in the opposite region of the distal FeS cluster [70]. Gutierrez-Sánchez et al. studied the reconstitution of the membrane-bound enzyme over gold electrodes with two different configurations characterized by AFM and electrochemical measurements [60]. In the first, the enzyme was attached through its lipid tail after the formation of a fBML over a gold electrode that was modified with a 4-aminothiophenol (4-APh) SAM. In this case, catalytic current for H_2_ oxidation was only measured by MET when adding 0.16 mm of methyl viologen (MV), because the Hase´s distal FeS cluster was facing the solution, not the electrode. This result also showed the existence of defects in the membrane allowing permeability of MV. The second configuration was obtained immobilizing the Hase together with liposomes and BioBeads (to remove detergent excess) in a single step over the SAM-modified gold electrode. The positive charges at the electrode surface oriented the immobilized Hase molecules by electrostatic interactions with the negatively charged region surrounding the distal FeS cluster, leaving the lipid tail towards the solution that allowed for the formation of a fBLM on the top of the Hase layer. With this last system, a clear catalytic current of H_2_ oxidation was observed by DET [60], as well as the generation of a proton gradient across the fBLM [71]. The AFM characterization indicated that the fBLM thickness was the expected one of approximately 5 nm [60], whereas the SEIRA studies confirmed the two different structural configurations that were obtained with these two reconstitution strategies [72].

**Figure 5:**
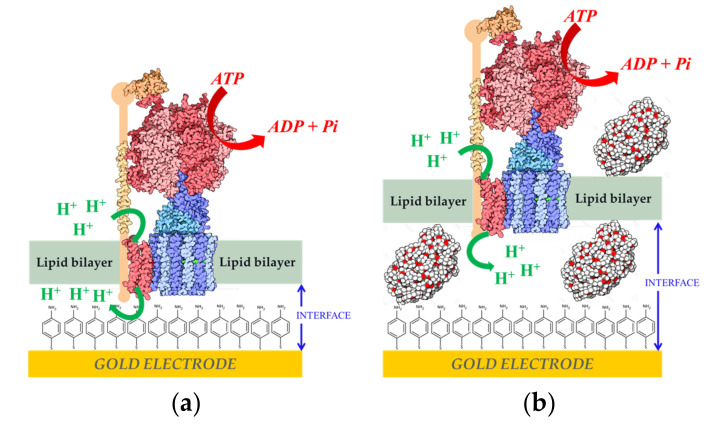
Adenosin triphosphate-synthase from *Escherichia coli* (ATPase) reconstitution in (**a**) fBLM (**b**) fBLM-polyethylene glycol (fBLM-PEG) over gold electrodes modified with a 4-APh self-assembled monolayer (SAM). Reprinted from [77] with permission from Elsevier.

Another step further was the coupling of the ability of this system to generate a proton gradient across a biomimetic membrane over an electrode with the activity of another membrane-bound enzyme, F_1_-F_0_ ATP-synthase from *Escherichia coli* (ATPase). This enzyme uses the proton gradient across the membrane as a driving force for the synthesis of adenosine triphosphate (ATP) [73,74,75]. ATPase was inserted into liposomes and a fBLM was formed by the fusion of the proteoliposomes over the NiFeSe Hase monolayer covalently attached to the electrode surface. In the presence of 500 µm of adenosine diphosphate (ADP) and phosphate in the solution, 40 µg of ATP was synthesized in 2 h [76]. The AFM images indicated that 30–40% of the surface was covered by enzyme, thus the amount of the ATPase on the gold surface was estimated to be around 350 ng cm^−2^. It was further reported that ATPase proteoliposomes could be directly fused over the gold electrode modified with the 4-APh SAM to form a fBLM. Two types of proteoliposomes were studied for ATP hydrolisis: with and without poly (ethylene glycol) 5000 MW (PEG) (Figure 5). AFM and electrochemical measurements indicated that more reproducible and stable results were obtained when PEG was included as spacer between the fBLM and the electrode surface. This improvement was attributed to an increase of the hydrophilic boundary, allowing for more translocation of protons across the fBLM [77].

A fBLM on gold electrodes modified with 4-APh has also been used to reconstitute the multienzimatic complex I (CpI) [78], which plays a fundamental role in the production of cellular energy. It contributes to the stabilization and maintenance of the transmembrane electrochemical potential difference necessary for ATP synthesis, transport, and mobility. Deficiencies in this enzyme can lead to some neurodegenerative diseases, such as Leber’s hereditary optic neuropathy, Parkinson’s disorders, and dystonia [79]. The hydrophilic peripheral subunits contain the prosthetic groups (FeS clusters and FMN) for NADH oxidation and electron transport [80], while the hydrophobic subunits inserted in the cell membrane part are involved in quinone reduction and charge translocation [81]. The system allowed for the electrochemical study of electron transfer and proton translocation by CpI. 2,3-dimethyl-1,4-naphthoquinone (DMN) was incorporated into the bilayer as electron acceptor from the enzyme after NADH oxidation and as redox mediator at the electrode. Without DMN in the system the catalytic current was negligible. The AFM study indicated the presence of protuberances of 6–8 nm coming out from the fBLM, which were attributed to the hydrophilic components of reconstituted CpI molecules [78]. In a later work, the two functions of CpI were monitored by SEIRA spectroscopy. It was found that changing the way of constructing the system affected the amide I/amide II infrared bands intensity ratio, which might indicate different orientations or arrangements of the enzyme on the electrode. The SEIRA experiments showed that CpI was preferably incorporated into the fBLM with the catalytic hydrophilic arm towards the solution, which made it catalytically active on NADH oxidation and translocation of protons, thus acidifying the electrode/fBLM interface [82].

## 4. Applications as Biosensors

The immobilization of membrane-bound enzymes on tailored electrodes that mimic their natural environment allows for enhancing the electrocatalytic properties by preserving their optimal structural integrity, as indicated in the previous section. In consequence, more sensitive and stable biosensors that are based on this type of enzymes can be developed using these strategies. Furthermore, the presence of a biomimetic membrane over the electrode can prevent the fouling of its surface by the medium or the non-desired signals due to redox-active interferents.

An early example of a biosensor based on a membrane-enzyme co-immobilized with a biomimetic membrane was reported by Kinnear and Monbouquette [83]. Membrane-bound fructose dehydrogenase (FDh) was reconstituted with a mix of two phospholipids (dioleoyl-L-phosphatidyl ethanolamine and dioleoyl-L-phosphatidyl choline) and its natural cofactor ubiquinone-6. Subsequently, it was deposited on a gold electrode modified with a mixed SAM of thiols. The SAM contained a hydrophobic long-chain thiol together with two polar short-chain ones, in order to facilitate both electrostatic and hydrophobic interactions with the charged groups of the phospholipids and the largely lipophilic FDh, respectively. The enzymatic electrode was studied for amperometric detection of fructose, in which the ubiquinone-6 co-immobilized in the mixed thiolate/phospholipid layer acted as redox mediator. The calibration curve was linear up to 5 mm fructose with a detection limit of 10 µm. The biosensor was highly selective by showing no appreciable response to other sugars, which is due to the specificity of FDh activity towards fructose. The fructose biosensor was tested in apple and orange juice with low interference by ascorbate, which was attributed to the blocking effect of the hydrophobic layer on the electrode. The storage stability of the biosensor improved in comparison to the FDh-modified electrode without the biomimetic membrane, which was due to the decreased leaching of the ubiquinone redox mediator.

Darder et al. also immobilized FDh to gold electrodes that were modified with a mixed SAM of hydrophobic and hydrophilic thiols in order to simultaneously provide electrostatic interactions with charged residues of the enzyme and hydrophobic interactions with its largely lipophilic regions [84]. Sodium hydroxymethyl ferrocene in solution was used as redox mediator. The detection limit for fructose was 20 µM and the lineal range reached up to 0.7 mm, having similarly low ascorbate interference, as reported by Kinnear and Monbouquette [83]. The mixed thiol SAM was also tested as an immobilization platform for another membrane-bound enzyme, D-gluconate dehydrogenase, giving good results for gluconate detection. On the other hand, no electrocatalytic responses were obtained when soluble redox enzymes, such us glucose oxidase (GOx) or horse radish peroxidase (HRP), were deposited on gold electrodes that were modified in the same way. The QCM measurements indicated that such enzymes did not immobilize on the surface, which indicated that the mixed SAM was only adequate for attaching lipophilic enzymes [84].

More recently, a FDh-based amperometric biosensor has been reported in which the membrane-bound enzyme was entrapped in liquid-crystalline lipidic cubic phase, an adequate matrix for lipophilic enzymes. The encapsulated FDh was then deposited on a glassy carbon electrode modified with single wall carbon nanotubes (SWCNTs) to allow for DET with the heme group of the enzyme. In this way, very large electroacatalytic currents were measured in the presence of fructose without requiring the addition of a redox mediator, thus obtaining a third-generation biosensor [85]. The linear range of the fructose biosensor was 1–10 mM. Furthermore, the operational stability of the enzymatic electrode was high, as no appreciable loss of the amperometric signal was observed during 10 h of continuous cycling. Indeed, this high stability of the immobilized FDh was attributed to the favorable environment that was provided by the lipidic matrix, preventing enzyme leaching or degradation.

**Figure 6:**
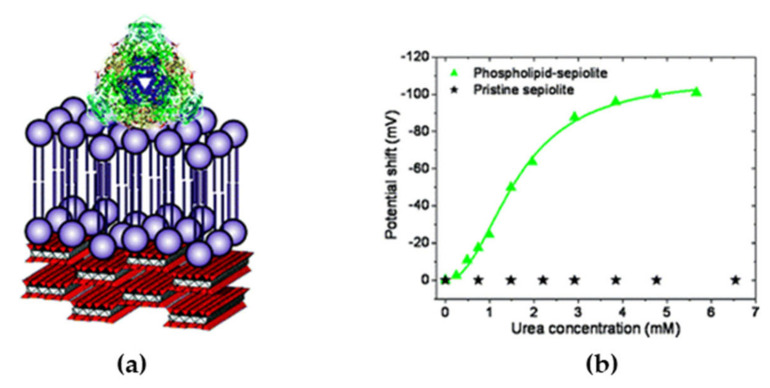
(**a**) Schematic representation of urease attached to a sBLM on sepiolite fiber. (**b**) Dependence on urea concentration of the potential shift of the redox probe as a result of pH increase due to the catalytic activity of urease immobilized on pristine and sBLM-modified sepiolite. Reproduced with permission from [88]. Copyright (2011) American Chemical Society.

Cholesterol oxidase (COx) is a flavoenzyme that catalyzes the oxidation of cholesterol while reducing O_2_ to H_2_O_2_ and presents a hydrophobic side chain that inserts into the lipid membrane in vivo [86]. The measurement of cholesterol levels in blood is a very important biomedical parameter for coronary heart diseases, arteriosclerosis, and cerebral thrombosis [87]. Wicklein et al. studied the immobilization of this enzyme on different biomimetic interfaces that were based on phosphatidyl choline (PC) assembled on to silicate sepiolite [88]. The best results were obtained when a PC bilayer was used, to which COx bound by inserting a side loop as it does in its natural environment. The activity of the enzyme in the presence of cholesterol was measured amperometrically by the oxidation of the H_2_O_2_ produced at the working electrode. The lineal range and sensitivity obtained were 0–5 µm and 154 mA M^−1^, respectively. The same PC bilayer on sepiolite was used for immobilizing urease, an enzyme that peripherally binds to the cellular membrane, to build an urea biosensor on top of a gold electrode modified with a SAM of 5,5’-dithiobis (2-nitrobenzoic acid) (Figure 6a). The electrochemical transduction was based on the pH change at the electrode surface due to the urea hydrolysis activity of the immobilized enzyme. The SAM on the electrode acted as potentiometric sensor, because its redox potential shifted 59 mV per pH unit (Figure 6b). Fast detection of urea was measured with high sensitivity (30.8 ± 0.7 V M^−1^) and low interference from ascorbic acid. The biosensor could be stored during at least six months without sensitivity loss, which indicated the excellent compatibility of the sepiolite/PC bilayer assembly with the enzyme [88].

Psychoios et al. reported another cholesterol biosensor based on COx immobilized on an electrode in a biomimetic configuration [89]. The enzyme was encapsulated in a lipid film that was polymerized over a matrix of ZnO nanowalls, which acted as transducer by providing a potentiometric signal related to the change of the double layer charge on their surface caused by the enzymatic reaction. The biosensor was tested in blood serum and urine, having a sensitivity of 57 mV per decade of cholesterol concentration, a broad logarithmic detection range of several orders of magnitude, a limit of detection of 0.7 µm, low interferences from albumin, and excellent storage stability.

**Figure 7:**
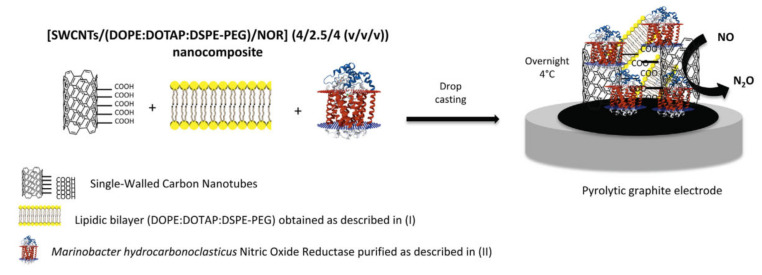
Scheme of nitric oxide biosensor construction. Reprinted from [90] with permission from Elsevier.

Very recently, Moura and co-workers have reported an interesting third-generation nitric oxide biosensor based on the membrane-bound enzyme nitric oxide reductase [90]. NO is involved in many biological processes, but it is very reactive, thus its quantification requires detection times between 5 and 15 s [91]. The biosensing device was formed by a mix of the enzyme, BLMs, and SWCNTs on a pyrolytic graphite electrode (Figure 7). The SWCNTs served as wires for DET from the electrode to the enzyme and were functionalized with carboxylic groups to favor electrostatic interactions with the predominantly positively charged phospholipids in the bilayer composition. A polyethylene glycol (PEG)-modified phospholipid was included to avoid liposome formation, while favoring the stabilization of the membrane-bound enzyme within the SWCNTs-BLM network. Square wave voltammetry showed DET of nitric oxide reductase and the peak current increased linearly with NO concentration in the 0.4–1.0 µm range. A limit of detection of 0.13 µm was measured, which is adequate for measuring NO evolution in biological processes. The storage stability was good with 83.5% of the initial response was retained after five weeks, which indicated sufficient binding between the three components that were deposited on the electrode that minimized leaching [90].

There are many studies of electrochemical biosensors that are based on transmembrane proteins reconstituted on phospholipid bilayers supported on electrodes, although the vast majority comprises ion-transport proteins or olfactory receptors, not enzymes [57]. However, a very interesting transmembrane enzyme for biosensing is ATP-synthase or ATPase. The measurement of adenosin triphosphate (ATP) concentration is of great interest for studying cell metabolism [92] and for the detection of microbial contamination on surfaces [93]. In order to develop a sensitive and versatile biosensor for ATP detection, the ATPase from *Escherichia coli* was reconstituted on a floating phospholipid bilayer over a gold electrode modified with a 4-APh SAM (Figure 5). The role of the thiol SAM was not only to favor the formation of the biomimetic membrane over the electrode, but also to serve as redox probe of pH changes at the electrode/fBLM interface after oxidation and dimerization [71]. In the presence of ATP in solution, the ATPase hydrolyzes the compound while translocating protons across the membrane. The potential of the redox probe on the gold surface is pH-dependent, thus by differential pulse voltammetry a positive shift of the peak potential was measured that was proportional to the logarithm of ATP concentration in the bulk solution in the 0.001–1 mM range. The potentiometric ATP biosensor was tested for monitoring the presence of microbial contamination. ATP extracted from *Escherichia coli* cultures with different cells concentration were analyzed with the biosensor obtaining an electrochemical signal proportional to the cells concentration with a time response of 5 min [77].

A very original electrochemical biosensor design has been reported for the detection of aldolase activity. In this case the biosensor does not integrate a membrane-bound enzyme; instead the catalytic activity of the soluble enzyme induces the formation of a tBLM on the electrode surface that decreases the amperometric response of the redox probe ferricyanide. Aldolase is a disease marker for cancer diagnosis and it catalyzes the reversible conversion of fructose-1,6-bisphosphate to glyceraldehyde 3-phosphate and dihydroxyacetone. Both products of the enzyme reaction have carbonyl groups that acted as linkers between the SAM thiol on the Au electrode and a PEG-phospholipid, thus driving the formation of a tBLM that blocked ferricyanide reduction at the electrode. The aldolase activity was analyzed over a broad linear detection range from 5 mU L^−1^ to 100 U L^−1^, measuring a 1 mU L^−1^ detection limit [94].

Liposomes can be also used to stabilize a membrane-bound enzyme on an electrode for improvement of biosensor performance. Besides providing a biomimetic environment to the enzyme, the liposomes give protection from proteases action and improve confinement on the electrode surface. Guan et al. developed an electrochemical biosensor for organophosphorus pesticides formed by layer-by-layer deposition of chitosan (a biocompatible polymer) and spherical shell liposomes containing acetylcholinesterase (AChE) [95]. AChE is a transmembrane enzyme that is part of the nervous system, in which it maintains the level of the neurotransmitter acetylcholine by catalyzing its hydrolysis into thiocholine [57]. Porins were added to the liposomes that were deposited on the electrode to allow the entrance of AChE substrate and pesticide inhibitor. Dichlorvos was used as a model pesticide for studying the biosensing properties of the enzymatic electrode. Amperometric transduction was performed by measuring the decrease of the oxidation current of the thiocholine product due to the inhibition of AChE by the action of the pesticide. The amperometric biosensor was quite sensitive to dichlorvos with a linear range of 0.25–10 µm and a detection limit of 0.9 ± 0.1 µg L^−1^. The storage stability of the biosensor was studied, retaining full activity after storing for 15 days, whereas, in comparison, the enzyme in solution lost 80% of its activity [95]. In a subsequent work, the same authors increased the sensitivity of the biosensor by including multiwalled carbon nanotubes in the layer-by-layer system to improve the electrochemical detection of thiocholine. The optimal construction involved six complete layers, in which the detection limit for dichlorvos was 0.68 ± 0.076 µg L^−1^. In addition, the storage stability improved by retaining full activity after 30 days [96].

## 5. Perspectives

The number of reported electrochemical biosensors based on membrane-bound enzymes is still quite low when compared to those based on soluble enzymes, even if, in nature, the amount of enzymes of the first kind that catalyze reactions of interest in sensing is large [3,5,57]. Therefore, we think that there is a great potential for increasing research in this particular field. A very important issue for this purpose is to develop reliable methods for immobilizing membrane-enzymes onto electrodes while maintaining their native structure.

Biomimetic chemistry comes to aid for affronting this challenge. In the last decades, there has been large progress on the formation and characterization of biomimetic lipid bilayers on electrodes for fundamental studies [8,19]. Besides stabilizing the membrane-bound enzyme, the lipid bilayer over the electrode might provide protection from surface fouling or from redox-active interferents. More studies should be done in this direction in order to obtain more stable and specific biosensors for analysis in complex samples. Although sBLMs and tBLMs have shown to be very valuable as model systems for fundamental studies of cell membrane processes [9,25,29,45,48], we think that fBLMs are more adequate for biosensor development. The presence of a water cushion in the interface between the fBLM and the electrode facilitates the insertion of transmembrane proteins in the lipid bilayer and gives higher flexibility and mobility within the biomimetic construction [77,78,82]. Moreover, the presence of an aqueous interphase facilitates charge transfer process across the membrane, which can help in coupling the biological recognition event to the electrochemical transductor [77,78]. Thus, we think that there is great potential for the development of biosensors with potentiometric or impedance detection. Not only BLMs are valid for providing a suitable biomimetic immobilization of membrane enzymes on electrodes. Other alternatives, such as formation of mixed SAMs with controlled hydrophobic/hydrophilic composition [83,84], entrapment in liquid-crystalline lipidic cubic phase [85], or deposition of liposomes on the electrode surface have been shown to be successful for different biosensor applications [95,96].

Several surface characterization techniques are now available for electrochemical research, such as AFM/STM, SERS, SEIRAS, and PM-IRRAS, which allow for the complete characterization of the biomimetic constructions and immobilized membrane enzymes on the electrode [8]. The use of these surface characterization techniques combined with the diverse panoply of existing electrochemical methods permits correlating the configuration of the immobilized membrane enzyme in the biomimetic construction with its electrocatalytical properties [7,69,72,82]. In our opinion, this kind of studies should, in the future, considerably aid the optimization of biosensors design based on biomimetic systems.
